# Quantification of Major Bacteria and Yeast Species in Kefir Consortia by Multiplex TaqMan qPCR

**DOI:** 10.3389/fmicb.2020.01291

**Published:** 2020-06-16

**Authors:** Fatemeh Nejati, Stefan Junne, Jens Kurreck, Peter Neubauer

**Affiliations:** ^1^Department of Bioprocess Engineering, Faculty III Process Sciences, Institute of Biotechnology, Technische Universität Berlin, Berlin, Germany; ^2^Department of Applied Biochemistry, Faculty III Process Sciences, Institute of Biotechnology, Technische Universität Berlin, Berlin, Germany

**Keywords:** TaqMan qPCR, milk kefir, quantification, microbial community, lactic acid bacteria, yeast, *Acetobacter*

## Abstract

Kefir grains are complex microbial systems of several groups of microorganisms. The identification and quantification of the microbial composition of milk kefirs was described in several studies, which provided an insight into the microbial consortia in this complex ecosystem. Nevertheless, the current methods for identification and quantification are not appropriate for deeper studies on kefir consortia, e.g., population dynamics and microbial interactions in kefir grains. This requires another sensitive and reliable quantitative method. Therefore, this study aims to develop multiplexed qPCR assays to specifically detect and quantify, as an example, several microorganisms of the milk kefir microbial community. Primer-probe sets, which target species-specific genes in six bacteria and five yeasts, were designed, and their sensitivity and specificity to the target species was analyzed in simplex as well as four multiplex qPCR assays. The self-designed multiplex assays were applied for the detection of target bacteria and yeast species in milk kefirs, in both, grain and beverage fractions. Detection of all target microorganisms in simplex and multiplex qPCR was achieved by good linearity, efficiency, repeatability and reproducibility in all assays. When the designed assays were applied on six kefirs, all target microorganisms were detected in different samples, but not all in one kefir sample. The two ubiquitous lactobacilli *Lactobacillus kefiranofaciens* and *Lb. kefiri* were present in all six kefirs studied, but were associated with different other yeasts and bacteria. Especially on the yeast community a significant diversity was observed. In general, multiplex TaqMan qPCR as developed here was proven to have high potential for specific identification of target microorganisms in kefir samples and for the first time, eleven target bacteria and yeasts of kefir microbiota were rapidly detected and quantified. This study, thus, provides a fast and reliable protocol for future studies on kefir and other similar microbial ecosystems.

## Introduction

Kefir is an ancient fermented milk beverage, which became very popular recently. Traditional kefir is produced by fermentation of milk with kefir grains, which is a protein-polysaccharide structure that contains a complex mixture of lactic acid bacteria (LAB), acetic acid bacteria (AAB), and yeasts. More than 50 microbial species were identified in different milk kefirs (Bourrie et al., 2016; Liu et al., 2019). It is supposed that several inter-microbial actions are responsible for the maintenance of the kefir grains’ integrity as well as their bio-functionalities as robust starter cultures (Nejati et al., 2020). The microbial composition of kefir grains can change due to several factors, such as the cultivation conditions (e.g., temperature, grain to milk ratio, milk source) and the geographical origin of kefir (Prado et al., 2015).

Several culture-dependent and -independent methods were applied to gain a deeper view into the microbial composition of kefir in the last decade. Accurate identification is essential to thoroughly understand the community functions and its proper industrial application. Culture-dependent methods enable the quantification of microbial species on the basis of colony forming units (CFU), although this method has several drawbacks; a long duration (especially for several slow growing species of lactobacilli such as *Lactobacillus (Lb.) kefiranofaciens* in kefir), a rather low reproducibility and an inability to differentiate accurately between bacterial strains (Hansen et al., 2018). Additionally, the accuracy is low since viable-but-non-culturable cells (VBNC) cannot be counted (Liu et al., 2018). PCR-denaturing gradient gel electrophoresis (PCR-DGGE) was the most common culture-independent method for the identification of kefir microbiota for many years (Chen et al., 2008). Later, however, studies proved that this method has a low accuracy when the composition of complex microbial ecosystems need to be investigated (Leite et al., 2012; Garofalo et al., 2015). Although next generation sequencing (NGS) techniques are powerful approaches when studying microbial community composition, they still suffer from misclassification at the species level (Winand et al., 2019). This might lead to a wrong identification of bacteria and yeasts that belong to a same genus. Additionally, concerns about technical pitfalls and potential biases were raised in literature when results were interpreted (Nilsson et al., 2019; Jian et al., 2020). Nevertheless, these techniques provided successfully a more detailed picture on the microbial composition of kefir, especially for low abundant species (Zamberi et al., 2016; Gao and Zhang, 2019). In order to study kefir in different aspects apart from the pure microbial composition, for example the generation of grains and the population changes in response to environmental conditions, the identification techniques, which have been applied so far are insufficient. The authors thus suppose that the development of a species-specific assay is a requirement to unravel the mysteries on the formation, integrity and functionality of kefir grain generation and knowledge-driven co-cultivation of species, which were isolated from kefir.

Quantitative real time polymerase chain reaction (qPCR), due to its high specificity and selectivity, is a qualified method for the detection of a specific microorganism in a microbial matrix. For qPCR, two reporter systems are commonly used: (i) intercalating dyes such as SYBR Green and (ii) fluorogenic hybridization oligoprobes, particularly 5′ exonuclease assays (also called TaqMan^TM^) (Smith and Osborn, 2009). While intercalating dyes bind non-specifically to all generated amplicons, dual-labeled oligoprobes of TaqMan assay anneal specifically to a target region, which guarantees specific detection (Smith and Osborn, 2009). In addition, application of TaqMan probes facilitates multiplexing as several differently labeled probes can be used simultaneously in the same assay (Duffy et al., 2013). High costs for labeling of each end of the probes with different dyes is in comparison to the use of intercalating dyes is compensated by higher selectivity and a reduced process time in multiplexed assays. Multiplex TaqMan qPCR recently appeared to be a very useful tool for the identification and quantification of different microbial communities (Farkas et al., 2017; Baudy et al., 2019; Enora et al., 2019; Lochman et al., 2019). Accordingly, this study aims to establish a multiplex qPCR approach for the simultaneous detection and quantification of eleven frequently reported bacteria and yeast species of milk kefirs. In order to achieve this, accuracy and precision of multiplexed qPCR assays were assessed toward target and non-target microorganisms, while the applicability was evaluated by quantification of the targeted species in six milk kefirs, in both grain and beverage fractions.

## Materials and Methods

### Microbial Strains and Genetic Materials

The bacterial and yeast strains used in this study are shown in Table 1. LAB strains were cultivated in De Man-Rogosa-Sharpe (MRS) broth (Carl Roth, Germany). The medium was supplemented with 10% filter-sterilized white table wine for cultivation of two strains of *Lb. kefiranofaciens* 5016 and 10550. *Acetobacter (A.) orientalis* and *A. fabarum* were cultivated in AAB broth [5 g L^–1^ yeast extract (Merck, Germany), 5 g L^–1^ bacto peptone (Merck, Germany), 5 g L^–1^ glucose (Merck, Germany), 1 g L^–1^ MgSO_4_ × 7 H_2_O (Sigma, Germany)] and yeasts were cultivated in YPG broth [10 g L^–1^ yeast extract, 20 g L^–1^ peptone (Merck, Germany), 20 g L^–1^ glucose]. All strains were incubated at 30°C under static aerobic conditions, except *Lb. kefiranofaciens* ssp., which were cultivated under severe oxygen-limited conditions (Anaerocult A^®^; Merck, Germany). The genomic DNA (gDNA) of bacteria and yeast species was extracted from exponentially growing cultures [24 h for all strains except *Lb. kefiranofaciens* ssp. and *Kazachtania (Kz.) turicensis*, which were cultivated between 4 and 5 days] by using a NucleoSpin^®^ Microbial DNA kit (MACHEREY-NAGEL, Germany). Quality and concentration of extracted gDNA was measured with a NanoDrop ND-1000 spectrophotometer (Peqlab Biotechnologie, Germany).

**TABLE 1:** Microorganisms, which were used in this study.

**Microorganism**	**Species**	**Strain name**	**Function**
Bacteria	*Lb. kefiranofaciens* ssp. *kefiranofaciens*	DSM 5016^T^	Target
	*Lb. kefiranofaciens* ssp. *kefirgranum*	DSM 10550^T^	Target
	*Lb. kefiri*	DSM 20587^T^	Target
	*A. orientalis*	FKG1^a^	Target
	*Ln. mesenteroides*	LG2-A^a^	Target
	*A. fabarum*	P3S1^a^	Target
	*Lc. lactis* ssp. *cremoris*	NZ9000	Target
	*Lc. lactis* ssp. *lactis*	IL1403	Target
	*Lb. helveticus*	DSM 20075^T^	Non-target
	*Lb. reutrei*	DSM 20016^T^	Non-target
	*Lb. paracasei*	DSM 20008	Non-target
	*Lb. parakefiri*	DSM 10551^T^	Non-target
	*Lb. plantarum*	SZ5^b^	Non-target
	*Weissella cibaria*	HY21^b^	Non-target
Yeasts	*Kz. turicensis*	DBVPG 7206^T^	Target
	*Kz. unispora*	DBVPG 6429	Target
	*Kl. marxianus*	DBVPG 6141	Target
	*S. cerevisiae*	DBVPG 10191	Target
	*D. anomalus*	DBVPG 10201	Target
	*Kz. exigua*	DBVPG 3191	Non-target

*^*T*^Type strain. ^*a*^Strains were isolated from kefir and identified by 16S sequencing, unpublished data. ^*b*^Strains were isolated and identified by 16S sequencing in a previous study (Taghi-Zadeh and Nejati, 2017).*

### Primers and TaqMan Probes Design and Verification

Primers and probes for multiplexed assays were designed using the Beacon Designer^TM^ software (v. 8.21, PREMIER Biosoft International, United States) after specific genes for each target species were selected previously. The criteria for the selection of target genes were: (i) species-specificity of the chosen gene/sequence as investigated via NCBI’s BLAST algorithm^1^, (ii) existence of only one copy of selected gene/sequence in each target species, and (iii) suitability of the selected sequence as provided by the Beacon Designer^TM^ software for designing of primers and probes (e.g., GC content of the sequence) to achieve higher design success rates, respectively. Parameters for the design of primer-probe sets in multiplexed assays (e.g., length, melting temperature and GC content of primers, probes and amplicons) were applied as defined by the default settings of the software. In order to increase the stability, specificity and sensitivity of the TaqMan probes, each probe contained four Locked Nucleic Acid^TM^ (LNA) bases. In Table 2, information is summarized about the target species, target genes in each species and all primer-probe set sequences, which were generated in this study.

**TABLE 2:** Information on target species and their primer-probe set sequences generated in this study in addition to qPCR assay set up.

**Organism**	**Genom accession number**	**Target gene**	**Primer/probe**	**Sequence (5′-3′)**	**Melting Tem. (°C)**	**Amplicon size (bp)**	**Assay (s)**
*Lb. kefiranofaciens*	AZGG00000000	DNA helicase RecG	Forward Reverse Probe^a^	GCAACAACCAAAGTATTGTA TAGCCGAAGAGGATCTAA **Q705**-ACC[+A]CA[+T]CA[+C]CA[+A]CTCTAA-**BHQ3**	60.1 59.8 69.0	118	1 and 2
*Lb. kefiri*	AYYV00000000	β-glucuronidase	Forward Reverse Probe	TCGCTTTCAAGCATTGAA CGAACTTCCCATTATCCATA **Cy5**-CAT[+C]AA[+G]CC[+A]AC[+A]GCAG-**BHQ3**	61.4 60.4 68.7	134	1 and 2
*A. orientalis*	AP018515.1	Coproporphyrinogen III oxidase	Forward Reverse Probe	CAGAGTATTACCCWCGCTTTA GGTGAAGGCAAAGTCTTG **TxRed**-CAA[+T]GT[+T]GC[+C]AC[+T]ATCGAG-**BHQ2**	62.7 62.1 68.0	137	1 and 2
*Ln. mesentreoides*	LAYU00000000	Mannose-6-phosphate isomerase	Forward Reverse Probe	CGAACCAACAACTTATCTATG CCTCATGTAGATCCTACCTTA **HEX**-TAC[+C]AC[+G]AT[+T]GT[+T]GACCA-**BHQ1**	60.3 61.2 69.4	107	1 and 2
*Lc. lactis*	NC_002662	Phosphotransferase system cellobiose-specific component IIC	Forward Reverse Probe	ACCTCTTGGACTTAATAACC CACGTACCCAAAGGTTAA **FAM**-CAC[+T]CA[+C]AA[+G]GT[+A]TCCGT-**BHQ1**	60.2 60.3 69.4	131	1
*A. fabarum*	NCXK01000006	Glutamate racemase	Forward Reverse Probe	CAGGCATTGGTGGATTAG TGGGAAAGAAGGGAGATAA **FAM**-ATC[+A]TC[+C]TG[+C]TC[+A]CCGTA-**BHQ1**	61.3 60.9 71.2	148	2
*Kz. turicensis*	PPOO01000000	Threonylcarbamoyl-AMP synthase	Forward Reverse Probe	CAAGGTTAAACCAGAATCAA GCAACTGTATCGTCTGTA **Cy5**-TTC[+T]CG[+C]CT[+A]AC[+T]CCGTA-**BHQ3**	59.5 59.8 69.4	138	3 and 4
*Kl. marxianus*	CM004405	Golgi apparatus membrane protein TVP38	Forward Reverse Probe	TCCTCGACAGTAATGATAA AGCACTCAATTCATCGTA **TxRed**-CTC[+C]TG[+A]TA[+G]AC[+C]GCTT-**BHQ2**	58.4 59.4 69.1	140	3 and 4
*D. anomalus*	MDSB00000000	Phosphoglycerate kinase	Forward Reverse Probe	GAGCAGACTGAGAAGTTC CGACCATAGAAGAGTGAG **FAM**-ATT[+G]AC[+C]GC[+T]CT[+T]GCT-**BHQ1**	60.5 59.6 68.9	100	3 and 4
*Kz. unispora*	PPON00000000	RNA polymerase II	Forward Reverse Probe	GTTGCATGGCAATCAAAA CGAAGACGCTCAAGAATA **HEX**-TCT[+T]CT[+T]CT[+T]CG[+G]CATCAA-**BHQ1**	60.7 60.2 68.4	101	3
*S. cerevisiae*	GCA_000146045	Golgi transport complex subunit COG6	Forward Reverse Probe	CGACAACAAATTGCTGAA CTCTCGAACATAACTCTGTA **HEX**-CAT[+C]CA[+G]TC[+G]CT[+A]TCCAAT-**BHQ1**	60.0 59.7 68.8	147	4

*^*a*^Each oligoprobe was labeled with a fluorescent dye (Q705, Quasar 705; Cy5, Cyanine 5; HEX, Hexachloro-fluorescein; TxRed, Texas red and FAM, 6-carboxyfluorescein) at 5′ end and corresponding quencher (Black Hole Quencher: BHQ3, BHQ2, and BHQ1) at 3′ end.*

Firstly, the specificity of primers, probes and amplicons was verified by performing a NCBI’s BLAST. Then, the amplicon sequences were checked against the whole corresponding microorganism genome using BLAST search. Next and prior to probe synthesis, the specificity of all primer pairs (synthesized by Sigma-Aldrich, Germany) was tested in simplex or multiplexed reactions using conventional PCR in order to investigate the production of expected amplicons of target species, in addition to the verification of absence of the products in non-target species. For this purpose, 10 μL (final volume) of a PCR mixture consisted of 2 μL Green GoTaq reaction buffer, 0.05 μL GoTaq DNA polymerase (5 U μL^–1^; Promega, Germany), 0.2 μL of dNTPs mix (each 10 mM), appropriate amounts of each primer to reach a final concentration of 1.2 μM in simplex PCR reactions or 0.8 μM in multiplex PCR, and 0.7 μL DNA template. Both, simplex and multiplex PCR were performed in an Eppendorf MasterCycler under the following cycling conditions: 95°C for 5 min; 35 cycles of 95°C for 30 s, 45°C for 30 s, and 72°C for 20 s; and a final extension of 5 min at 72°C. PCR products were analyzed on 2% agarose gels (Roth, Germany) containing the GelRed^TM^ dye (Biotium, Inc., United States).

Then, each pair of primers was used separately in qPCR runs with or without DNA template, in order to investigate the amplification of a single product in target microorganisms and the absence of primer-dimers. For this experiment, 2x SYBR^®^ Green Master Mix (Bio-Rad, Germany) was used in a 20 μL batch containing 400 nM of each of the primers. The qPCR program was initialized by 95°C for 5 min, continued by 40 cycles of 95°C for 15 s and 60°C for 30 s, followed by a melting curve to verify amplification specificity and the absence of primer dimers. qPCR was performed with a Bio-Rad CFX96 real-time PCR thermocycler by applying the “all channels” reading mode in 96-microwell clear unskirted plates (Biozym, Germany) that were sealed using optical adhesive films (Bio-Rad, Germany).

LNA^TM^ substituted TaqMan^®^ probes were obtained from Merck (Haverhill, United Kingdom); they contained of the fluorescent reporter dye on the 5′- end and the non-fluorescent quencher on the 3′-end. Probes for six bacteria and five yeast species were designed for the application in two distinct multiplexed assays, due to the limitation of qPCR thermocycler channels. These four experimental designs are presented in Table 2, assays 1 and 2 as fiveplex and assays 3 and 4 as fourplex set up. According to this, bacterial species *Lb. kefiranofaciens, Lb. kefiri, Leuconostoc (Ln.) mesenteroides* and *A. orientalis* were analyzed in assays 1 and 2, while *Lacococcus* (*Lc) lactis* and *A. fabarum* were analyzed only in assay 1 and assay 2. Similarly, yeast species *Kazachstania* (*Kz.) turicensis, Kluyveromayces (Kl.) marxianus* and *Dekkera (D.) anomalus* were analyzed in assays 3 and 4, while *Kz. unispora* and *Saccharomyces (S.) cerevisiae* were analyzed only in assay 3 and assay 4. Reporter dyes of each probe are shown in Table 2.

### Evaluation of Primers-Probe Sets and Assays

#### Analysis of Primers’ and Probes’ Specificity in Simplex qPCR

For analyzing the specificity of primer-probe sets in simplex reactions, each test comprised of six DNA standard concentrations from the target strain in addition to obligatory no-template controls (NTC). All analyses were performed in triplicate. The total reaction volume of 20 μL consisted of 2 μL gDNA (or ddH_2_O), 10 μL 2 × SsoAdvanced^TM^ Universal Probes Supermix (Bio-Rad, Germany), primers and probe, in order to reach a final concentration of 400 nM for each primer and 200 nM for the probe, and 7.52 μL of RNase-free water, respectively. The qPCR program covered an initial DNA polymerase activation step of 3 min at 95°C, 40 cycles of denaturation for 15 s at 95°C, and hybridization and extension for 30 s at 60°C, respectively. Fluorescence intensity was measured at the end of each cycle with the CFX Manager^TM^ software v3.1 (Bio-Rad). Finally, standard curves were created.

#### Evaluation of Multiplexed Assays

A complete microbial genomic (CMG) pool was prepared by mixing equal quantities of gDNA of all eleven target microorganisms. This CMG pool was then serially diluted 10-fold for the use as DNA template during the generation of data for standard curves in multiplexed qPCR assays. As outlined in Section “Evaluation of Primers-Probe Sets and Assays,” eleven target microorganisms were analyzed in four multiplexed assays (Table 2). In each assay, CMG was applied as DNA template, however, only primers and probes of the distinct target microorganism of that assay were added to the qPCR reaction. The total reaction volume of 20 μL consisted of 2 μL template (or ddH_2_O) and 18 μL master mix that contained 10 μL of 2 × iQ^TM^ Multiplex Powermix (Bio-Rad, Germany), and primer-probe sets to reach the final concentration of 300 nM of each primer, and 200 nM of each probe, respectively. The qPCR program covered an initial DNA polymerase activation step of 3 min at 95°C, 45 cycles of denaturation for 12 s at 95°C, and hybridization and extension for 45 s at 60°C, respectively. Fluorescence signals were detected in all channels; standard curves were plotted with the CFX Manager^TM^ software v3.1 (Bio-Rad).

#### Creation of Standards Curves

Standard curves were created by plotting the Cq against the log_10_ input genome copy number. The copy number of gDNA was calculated with formula (1)^2^ :

(1) $$\text{Number of copies = (amount of DNA, ng × }{\text{10}}^{\text{23}}\text{)/ (genome size, bp × }{\text{10}}^{\text{9}}\text{ × 660)}$$

The genome sizes used for calculations were 2257141, 2501983, 2036093, 3214967, 3095430, 2365589, 12134345, 12784682, 10776003, 14202998, 12323254, and 14202998 bp for *Lb. kefiranofacins* 5016, *Lb. kefiri* 20587, *Ln. mesenteroides* LBE16, *A. orientalis* FAN1, *A. fabarum* OG2, *Lc. lactis* IL1406, *S. cerevisiae* ySR128, *D. anomalous* YV396, *Kl. marxianus* LHW-O, *Kz. unispora* NRRL Y-1556 and *Kz. turicensis* NRRL Y-48834, respectively^3^. In simplex qPCR runs, gDNA of each microorganism was serially diluted 10-fold in water to final concentrations between 10^7^ and 10^1^ genome copies per μL. In multiplexed assays, CMG was serially diluted 10-fold to final concentrations between 10^5^ and 10^1^ genome copies per μL. The simplex and multiplex assays were evaluated based on their correlation coefficient (R^2^), slope and efficiency [*E* = (10^(–1/slope)^ − 1)] for standard curves, which were calculated by the software. Intra-assay repeatability was evaluated with the coefficient of variations (CV%) based on Cq-values; it was calculated for various concentrations from standard curves in replicate samples in the same PCR run. The CV% was also used to estimate inter-assay reproducibility when calculated for at least three independent PCR runs. The limit of detection (LOD) was defined as the Cq-value on the standard curve corresponding to 3 PFUs, while the limit of quantification (LOQ) was the concentration (copies reaction^–1^) corresponding to the LOD (Baudy et al., 2019).

### Quantification of Targeted Bacteria and Yeasts in Kefir Samples

#### Evaluation of DNA Extraction Kits

As DNA extraction can be a critical step in studies of microbial communities, we first compared the yield and quality of the DNA extracted from grains with two DNA extraction kits, either DNeasy PowerSoil Pro or DNeasy PowerBiofilm (Qiagen, Germany), each containing different bead systems. Two hundred milligram of grains from each of the two different home-made kefirs (PN and FN) were used for this investigation. DNA extraction was performed according to the manufacturer’s guidelines except that a MM400 Mixer Mill (Retsch, Germany) was applied for bead beating (10 min at 30 Hz) instead of TissueLyser. The quality of extracted DNA was measured using a spectrophotometer (NanoDrop ND-1000, Peqlab Biotechnologie, Germany).

#### Analysis of Targeted Species in Kefir Samples

Samples from six milk kefirs were analyzed in this study; four kefirs from home-made kefirs in Berlin (Germany, denoted as PN, FN, and BK) and Umbria (Italy, denoted as EK), as well as two samples from commercial kefirs (Primal Life UG, Berlin, Germany, denoted as CKM and CMC). Before analysis, 10 g grains of each kefir were added to 40 mL cow milk (Arla, 1.5% fat) and after 24 h fermentation at 25°C, with few occasionally shaking, samples were applied to DNA extraction. In all cases, the DNA was extracted from kefir beverages and kefir grains separately with the DNeasy PowerSoil Pro kit. In case of the beverage fraction, 1.8 mL of 24 h-fermented kefir was used as sample, which was treated according to the manufacturer’s protocol. Two grams of grains were washed by stirring 3 times in 50 mL of 0.85% NaCl, each for 15 min. Then, 0.2 g of grains were applied to DNA extraction.

As template, 2 μL of extracted DNA were separately used in qPCR analysis (as described in “Analysis of Targeted Species in Kefir Samples”). In each qPCR plate, three different concentrations of ten-fold serially diluted CMG (as described in “Analysis of Targeted Species in Kefir Samples”) in addition to NTC controls were applied. Reactions were rated as positive if all triplicates showed a Cq and if the average Cq was below the LOD of the respective microorganisms, otherwise it was rated as negative. After analysis, the number of each target species was extrapolated to 1 g of kefir grains or 1 mL of kefir beverage.

## Results and Discussion

Specific detection of *Lb. kefiranofaciens* in kefir with 16s rDNA-based TaqMan probes was reported by Kim et al. (2015b); however, this report describes the simultaneous analysis of eleven microorganisms in kefir samples for first time. These eleven microbial targets were chosen on the basis of several previous reports about the microbial composition of milk kefir (Garofalo et al., 2015; Blasche et al., 2019; Gao and Zhang, 2019). As an example, in kefirs studied by Blasche et al. (2019), *Lb. Kefiranofaciens, Lb. kefiri, Lc. lactis, Ln. mesenteroides* and *Acetobacter* spp. accounted for more than 95% of the kefir community.

Identification and quantification of different microbial communities is an important topic in microbiology. A development of sensitive and reliable quantification methods are needed in order to answer questions about microbial communities, for example how a population will respond to intrinsic or extrinsic factors and/or how these changes affect the quality of the final products. Kefir grains are complex microbial communities, hence, so far, artificially developed kefir starters for industrial applications did not result in the production of authentic final products. This is mainly due to a lack of knowledge on the exact microbial composition of natural starters, their functions, and their interaction with each other, respectively. Kim et al. (2015a) developed group-specific primers to quantify several groups of bacteria (e.g., *Lactobacilli, Lactococci, Streptococci, Enterococci*) and yeasts (e.g., *Candida, Saccharomyces*) in milk kefir with qPCR, however, a specific detection of defined target species in kefir has rarely been performed. The main goals of the current study were to highlight the potential of qPCR for fast detection and multiplex quantification of several target microorganisms in milk kefir as an example of a complex microbial community and to provide a sensitive and fast method for further studies on milk kefir.

### Target Genes Selection

A 16S rDNA in bacteria and 26S rDNA and ITS region in yeasts were commonly used for the identification and differentiation of microbial species in DGGE-PCR, high throughput sequencing (HTS) and qPCR techniques (Kim et al., 2015b; Korsak et al., 2015; Gao and Zhang, 2019; Okai et al., 2019). Nevertheless, as the varying parts of these regions are not long enough for generating high-rank primer-probe sets for multiplexing, other specific genes in each species were selected in this study.

**FIGURE 1:**
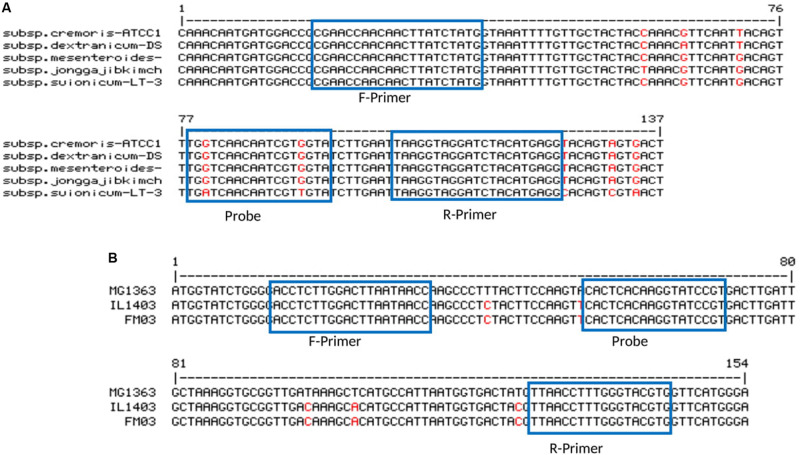
Primer-probe stets for panel **(A)** *Ln. mesenteroides* (ssp. *cremoris* strain ATCC 19254, ssp. *dextranicum* strain DSM20484, ssp. *mesenteroides* ATCC 8293, ssp. *jonggajibkimchii* strain DRC1506 and ssp. *suionicum* strain LT-38) and **(B)** *Lc. lactis* (ssp. *cremoris* strain MG1363, ssp. *lactic* strain IL1403 and ssp. *lactis* bv. diacetylactis strain FM03).

For some of the target species, either more than one subspecies was found in kefir microbiota or subspecies were not clarified. In order to be more specific, for *Lb. kefiranofaciens*, two subspecies *Lb. kefiranofaciens* and *Lb. kefirgranums* were isolated from milk kefir samples (Vancanneyt et al., 2004; Magalhães et al., 2010b), while *Lc. lactis* and *Ln. mesenteroides* subspecies were not defined in many studies. This can be possibly due to the poor discrimination power of 16S rDNA for subspecies differentiation. Accordingly, here we attempted to generate primer-probe sets that are able to be amplified in all subspecies for the target species. The attempt only failed for *Ln. mesenteroides*, as no region was found, which was long enough and suitable for the design of primer-probe sets that included all subspecies. Thus, *Ln. mesenteroides* ssp. *suionicum* was excluded (Figure 1). However, Jeon et al. (2017) proposed that *Ln. mesenteroides* ssp. *suionicum* should be reclassified as a novel species of the genus *Leuconostoc*, and not as a subspecies of *Ln. leuconostoc*, as it is genetically distant to other subspecies of *Ln. mesenteroides* according to the results of whole-genome-based taxonomic methods. *Lc. lactis* has two subspecies; *Lc. lactis* ssp. *lactis* and *Lc. lactis* ssp. *cremoris*, and one biovar; *Lc. lactis* ssp. *lactis* bv. *diacetlactis*; all of them are dairy products-related. The designed primer-probe set detect all of them (Figure 1). In *Acetobacter* group, the species *A. fabarum* and *A. lovaniensis* have a 99.79% identity in their 16S rDNA sequence (NR_042678 and NR_040832), which makes it difficult to discriminate them based on 16S rDNA sequencing. In several kefir studies, the detection of one of them was reported (Magalhães et al., 2010a; Blasche et al., 2019). The primers-probe set designed in this study can specifically detect *A. fabarum*, but does not recognize *A. lovaniensis*.

### Evaluation of Primer-Probe Sets Specificity

The specificity of designed primer-probe sets toward the target species was confirmed by bioinformatic analysis (NCBI’s BLAST). Next, the production of amplicons with the expected sizes was shown by conventional PCR experiments. No cross-reactivity was observed with non-target templates (Supplementary Figure S1). In addition to experiments with the target strains shown in Table 1, the specificity of primer sets was tested against several isolates, including two of *Lb. kfiranofaciens*, five of *Lb. kefiri*, four of *Ln. mesenteroides* and four of *A. orientalis* (data not shown). *Lb. helveticus, Lb. reuteri, Lb. paracasei, Lb. parakefiri, Lb. plantarum* and *Kz. exigua*, which were indicated as kefir microbiota in different studies (Bourrie et al., 2016). The absence of the amplification product in multiplex conventional PCR verified the specificity of the designed primer sets to the target microorganisms (Supplementary Figure S1).

Formation of primer dimers or unwanted PCR products through a non-specific binding of primers reduced significantly the sensitivity of qPCR analysis. Therefore, the formation of such products was investigated in this study. The melting curve analysis showed no primer dimer formation in all primer pairs (Supplementary Figure S2A). In addition, production of a single melting peak for each primer set verified the high specificity of the designed primer pairs toward target species (Supplementary Figures S2B,C for bacteria and yeast target species).

### Performance of Simplex and Multiplexed qPCR Assays

For all primer-probe sets, the fluorescence signal reached the threshold line only when the target microorganism was included in the reaction, which indicate all newly designed primer-probe sets have a high selectivity to the target species, both in simplex and multiplex qPCR assays. The correlation coefficient (R^2^), slop and efficiency (E) of standard curves in simplex and multiplex assays for the target microorganisms are shown in Table 3 (Additional Information in Supplementary Figures S3, S4 for simplex qPCR, and Supplementary Figures S5, S6 for multiplexed assays). The R^2^-values, ranging from 0.984 to 1.000, indicate a linear correspondence between the logarithmic genome copy number and their Cq-values. The efficiency (E), as one of the most important indicators of the performance of a qPCR assay, was between 86.4 and 104.7% in simplex, and between 90.6 and 103.4% in multiplex assays, which proves a good performance of the primer-probe sets (Baudy et al., 2019; Lochman et al., 2019). In all four assays, PCR probes appeared to be very specific and no loss of activity was observed upon multiplexing. The difference in R^2^, E and the slope for each target microorganism was negligible between simplex and multiplex assays. The intra-assay repeatability, inter-assay reproducibility, LOD and LOQ for each primer set are summarized in Table 4. The coefficients of variation (CV) were less than 3% and 4% for intra and inter-assay, respectively. According to the dilutions used in qPCR assays, the defined LOQs in multiplex assays were from 6 copies for *S. cerevisiae* to 75 copies for *Lb. kefiri* per qPCR reaction. These data demonstrate that the all qPCR assays had very good performances for the target species. Thus, multiplex qPCR was used in subsequent analysis on real kefir samples.

**TABLE 3:** Performance of primer-probe sets designed in this study in simplex and multiplex assays for target microorganisms.

**Target microorganism**	**Simplex qPCR**			**Multiplex qPCR**											
				**Assay 1**			**Assay 2**			**Assay 3**			**Assay 4**		

	**R^2^**	**E (%)**	**Slope^a^**	**R^2^**	**E (%)**	**Slope^a^**	**R^2^**	**E (%)**	**Slope^a^**	**R^2^**	**E (%)**	**Slope^a^**	**R^2^**	**E (%)**	**Slope^a^**
*Lb. kefiranofaciens*	0.999	94.9	3.456	0.998	99.0	3.346	0.997	103.4	3.242	−	−	−	−	−	−
*Lb. kefiri*	1.000	102.2	3.270	0.998	99.4	3.337	0.999	99.2	3.341	−	−	−	−	−	−
*A. orientalis*	0.999	89.1	3.613	0.999	100.0	3.322	0.997	97.1	3.394	−	−	−	−	−	−
*Ln. mesenteroides*	1.000	95.5	3.409	0.993	100.2	3.316	0.989	98.6	3.356	−	−	−	−	−	−
*Lc. lactis*	0.997	86.4	3.696	0.990	98.9	3.347	−	−	−	−	−	−	−	−	−
*A. fabarum*	0.997	93.0	3.502	−	−	−	0.995	101.7	3.281	−	−	−	−	−	−
*Kz. turicensis*	0.994	91.3	3.549	−	−	−	−	−	−	0.997	91.3	3.550	0.997	90.6	3.570
*Kz. unispora*	0.996	93.9	3.477	−	−	−	−	−	−	0.990	94.6	3.458	−	−	−
*Kl. marxianus*	0.996	88.4	3.636	−	−	−	−	−	−	0.997	100.1	3.319	0.998	96.5	3.408
*S. cervisiae*	0.997	104.7	3.214	−	−	−	−	−	−	−	−	−	0.997	98.1	3.369
*D. anomalus*	0.998	90.7	3.568	−	−	−	−	−	−	0.994	102.0	3.276	0.997	102.3	3.267

*^*a*^The slopes are negative values.*

**TABLE 4:** Intra- and inter-assay repeatability and reproducibility, LOD and LOQ of the quantification of eleven kefir-related microorganisms using multiplexed qPCR assays.

**Primer-probe set**	**DNA (copies reaction^–1^)**	**Intra-assay repeatability**			**Inter-assay-reproducibility**			**LOD (Cq)**	**LOQ (copies reaction^–1^)**
				
		**Mean-crossing point (Cq ± SD)**		**CV (%)**	**Mean-crossing point (Cq ± SD)**		**CV (%)**		
*Lb. kefiranofaciens*	5.44E + 05 5.44E + 03 5.44E + 02 5.44E + 01	18.71 25.17 28.63 32.11	0.26 0.09 0.36 0.17	1.40 0.36 1.25 0.54	18.46 25.07 28.26 31.92	0.29 0.32 0.52 0.54	1.56 1.27 1.85 1.68	32.11	5.44E + 01
*Lb. kefiri*	7.44E + 05 7.44E + 03 7.44E + 02 7.44E + 01	18.21 24.73 28.15 31.45	0.05 0.11 0.30 0.28	0.27 0.46 1.05 0.89	18.37 25.06 28.37 31.53	0.57 0.68 0.30 0.38	3.11 2.73 1.07 1.19	31.45	7.44E + 01
*A. orientalis*	2.76E + 04 2.76E + 02 2.76E + 01	22.32 29.02 32.33	0.17 0.41 0.10	0.76 1.41 0.31	23.07 29.70 32.98	0.61 0.55 0.92	2.63 1.84 2.78	32.33	2.76E + 01
*Ln. mesentreoides*	1.92E + 04 1.92E + 03 1.92E + 02	26.11 29.69 33.13	0.14 0.21 0.01	0.54 0.72 0.05	26.56 33.42 36.72	0.44 0.34 0.74	1.66 1.03 2.01	36.19	1.92E + 01
*Lc. lactis*	5.51E + 04 5.51E + 02 5.51E + 01	21.79 28.52 31.28	0.15 0.10 0.47	0.69 0.35 1.51	21.63 28.22 31.21	0.22 0.27 0.35	1.00 0.97 1.11	31.28	2.75E + 01
*A. fabarum*	1.73E + 05 1.73E + 03 1.73E + 02	20.01 26.18 29.30	0.26 0.23 0.07	1.30 0.88 0.25	20.35 27.06 29.87	0.44 0.88 0.81	2.18 3.25 2.70	33.33	1.73E + 01
*Kz. turicensis*	1.75E + 05 1.75E + 03 1.75E + 02	21.87 28.95 32.41	0.19 0.32 0.35	0.89 1.11 1.09	21.57 28.57 32.10	0.38 0.59 0.35	1.76 2.08 1.10	35.21	1.75E + 01
*Kl. marxianus*	5.25E + 04 5.25E + 02 5.25E + 01	22.42 29.22 32.27	0.07 0.03 0.15	0.31 0.11 0.46	22.82 29.67 32.29	0.40 0.36 0.02	1.77 1.22 0.07	32.27	5.25E + 01
*D. anomalus*	1.21E + 05 1.21E + 03 1.21E + 02	19.44 26.21 28.98	0.16 0.05 0.17	0.80 0.20 0.58	19.49 26.31 29.50	0.11 0.24 0.73	0.58 0.91 2.47	33.02	1.21E + 01
*Kz. unispora*	3.08E + 04 3.08E + 02 3.08E + 01	25.68 32.71 35.96	0.21 0.48 0.76	0.82 1.46 2.13	24.79 31.43 34.86	0.56 0.75 0.96	2.26 2.39 2.74	35.96	3.08E + 01
*S. cerevisiae*	6.02E + 04 6.02E + 02 6.02E + 01	22.01 28.84 32.35	0.04 0.05 0.42	0.20 0.18 1.32	22.79 29.86 32.70	0.68 0.88 0.68	2.99 2.95 2.08	35.42	6.02E + 00

*Cq, Cycle of quantification; CV, Coefficient of variation; SD, Standard deviation.*

### Analysis of Kefir Samples

#### Effect of DNA Extraction Kit

DNA extraction methods have shown to play a substantial role when the microbial composition of different communities were investigated (Ketchum et al., 2018; Vaidya et al., 2018). Here, gDNA from two kefir grain samples (PN and FN) was extracted by using the DNeasy PowerSoil Pro or DNeasy PowerBiofilm kits. These two kits benefit from different bead materials with various sizes (proprietary to manufacturer), which might impact DNA extraction yields. It was observed that the DNA extracted with the DNeasy PowerSoil Pro kit had a higher yield (21.1 ng μL^–1^) in comparison to the DNeasy PowerBiofilm kit (7.9 ng μL^–1^) for PN grain and 16.5 ng μL^–1^ compared to 5.1 ng μL^–1^ for FN grain, and relatively better purity (A260/230; 1.89 compared to 1.92 for PN grain, and 2.28 compared to 2.62 for FN grain) (Supplementary Table S1). Therefore, the DNeasy PowerSoil Pro kit was used for extraction of DNA.

#### Abundance of Target Bacteria and Yeasts Species in Kefir Samples

**FIGURE 2:**
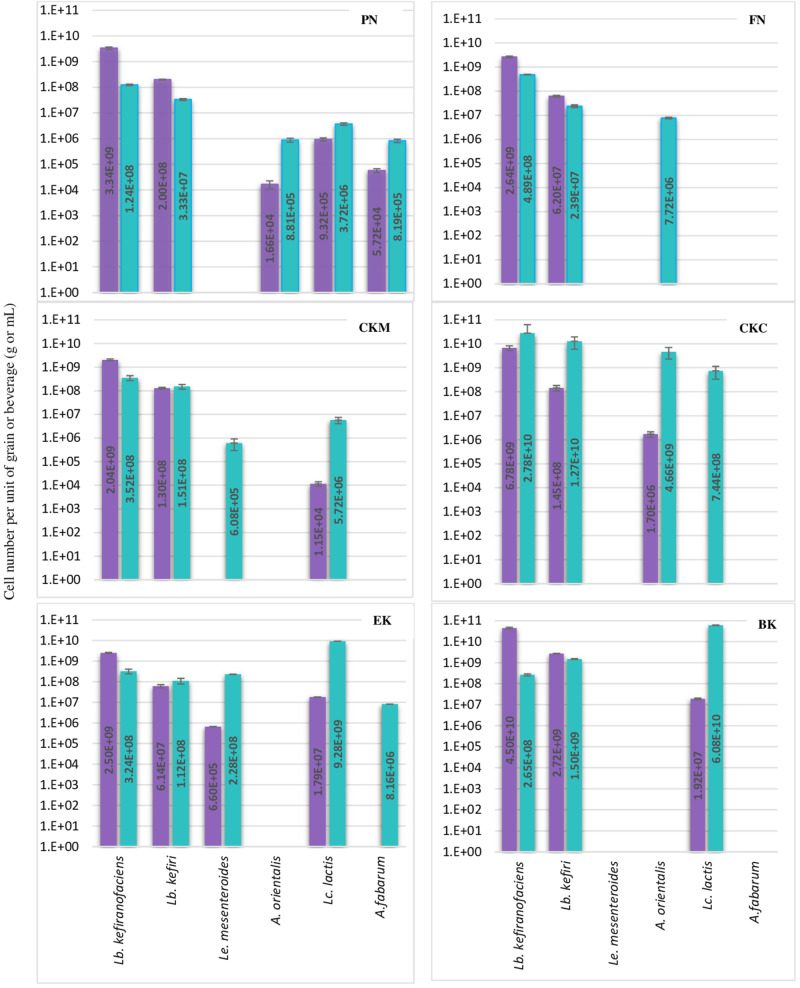
Number of each six bacteria species per g or mL of kefir grain (purple bar) or beverage fraction (green bar) of six milk kefirs. Numbers represent the mean of triplicate measurements. Error bars represent the standard deviation (SD).

**FIGURE 3:**
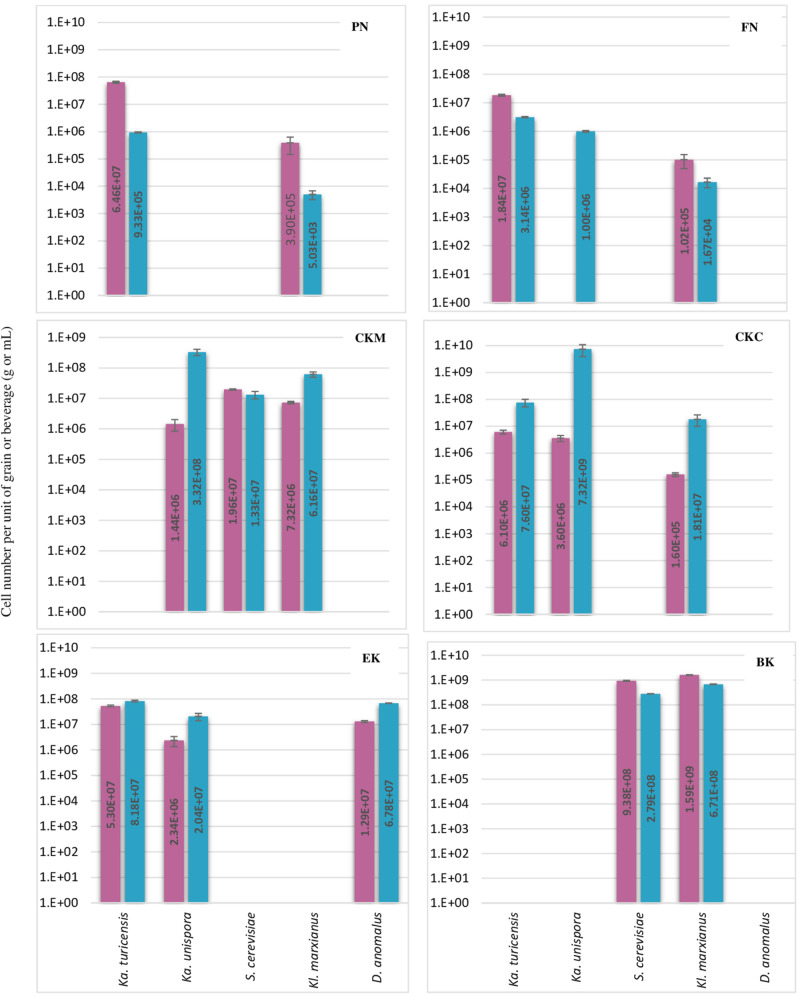
Number of each five yeast species per g or mL of kefir grain (red bar) or beverage fraction (blue bar) of six milk kefirs. Numbers represent the mean of triplicate measurements. Error bars represent the standard deviation (SD).

The abundance of eleven bacteria and yeast species in six milk kefirs was quantified by the previously described assays. The findings of this analysis are shown in Figures 2, 3 for bacteria and yeast communities. In general, all the kefir samples contained a minimum 2.17E + 09 and 1.63E + 08 number of identified bacteria, and 9.86E + 06 and 9.38E + 05 number of identified yeasts per unit of grain and beverage fraction (g or mL), respectively. These findings confirm data from previous studies (Guzel-Seydim et al., 2005), although it may be considered that not all microorganisms of the kefir microbiota were quantified in the present study.

*Lactobacillus kefiranofaciens* and *Lb. kefiri* strains were detected in all kefir samples. They constituted the major number of bacteria in grains and appeared in high numbers in kefir beverages (a minimum of 1.0E + 08 of *Lb. kefiranofaciens* and 1.0E + 07 of *Lb. kefiri* in mL of kefir beverage). The presence of the other four bacteria, i.e., *Lc. lactis, A. orientalis, A. fabarum* and *Ln. mesenteroides*, varied among the different kefirs. They were detected in the beverage fraction and not in the grain fraction in some samples (*A. orientalis* in FN, *A. fabarum* in EK, *Lc. lactis* in CKC and *Ln. mesenteroides* in CKM). Occasionally, in some beverage fractions, *Lc. lactis* appeared as dominant species, such as in kefirs EK and BK: the portion of identified bacteria constituted of *Lc. lactis* by 93.2% and 97.1%, while *Lb. kefiranofaciens* and *Lb. kefiri* were still dominant in the beverage fractions of FM and CKM kefirs, in which they represented more than 98% of the identified bacteria. *Ln. mesenteroides* was only detected in one of the kefir grains (EK) with a low percentage (0.02%) and did not exceed more that 2.2% of the total identified bacteria in the beverage fraction. Kefirs’ microbiota can differ with respect to *Acetobacter* species: although present in most samples, they were abundant in kefir BK. As it is believed that *Acetobacter* spp. support *Lactobacilli* growth by oxygen consumption and acetic acid release (Dong et al., 2018), it is possible that other *Acetobacter* species like *A. okinawensis* and *A. syzygii* are active with a similar function in BK kefir, which requires further investigations.

Results concerning the presence and abundance of specific species in kefir differ a lot among various studies, which is due to either differences in kefir samples or the role of different methods for the identification of microbiota (Nejati et al., 2020). As a result, a meaningful comparison of results among different studies are not easily feasible. For example, as mentioned above, *Lb. kefiranofaciens* and *Lb. kefiri* appeared to high amounts in all kefir beverage fractions in the current study, while Gao and Zhang (2019) reported them as a minority in five kefir beverage fractions using high-throughput Illumina sequencing technique. In general, our observations on the diversity of dominant bacteria in kefir beverages are closer to the findings of Korsak et al. (2015) who investigated microbial diversity of five kefirs by 16S rDNA pyrosequencing.

Regarding the yeast composition, a high variation among the five analyzed species was found within the six kefirs (Figure 3). According to the observations in this study, not all five yeast species were detected in one kefir sample, but it appeared that each kefir sample hosted between two and three of them. Among the five yeast species, *Kz. turicenis, Kl. marxianus, S. cerevisiae* and *D. anomalus*, were always detected in both, the grain and beverage fractions. This characteristic, however, seems to be strain specific for *Kz. unispora*, as well as for non-lactobacilli species, i.e., *Lc. lactis, A. fabarum, A. orientalis* and *Ln. mesenteroides*.

The yeast community of kefir has been found to be as complex as the bacterial community (Liu et al., 2019; Zhimo et al., 2020). Gao and Zhang (2019) analyzed five kefirs and found *Kazachstania*, *Kluyveromyces*, and *Saccharomyces* as the major genera, and *Kazachstania* species were found to be as most abundant in both grains and beverages. In *Kazachstania*, it seems that at least three species (*Kz. turicensis, Kz. Unispora*, *and Kz. exigua*) contribute to the yeast community of many milk kefirs (Garofalo et al., 2015; Blasche et al., 2019; Gao and Zhang, 2019). *Kz. unispora* has been isolated from kefir more often than the other two species. Reports on isolation of *Kz. turicensis* from kefir was not as frequent as *Kz. unispora*, until next generation sequencing methods were applied recently. In this study, *Kz. turicensis* and *Kz. unispora* each were found in four out of six kefirs. Garofalo et al. (2015) observed that both species were found as a minority in milk kefirs. *Kl. marxianus* and *S. cerevisiae*, individually or together, were frequently identified as absolute dominant yeasts in milk kefir samples (Leite et al., 2012; Kalamaki and Angelidis, 2017; Zhimo et al., 2020); however, in this study, *Kazachstania* species appeared in higher numbers than *Kl. marxianus* and *S. cerevisiae* in three kefirs PN, FN and CKC and in kefir EK none of the *Kl. marxianus* and *S. cerevisiae* were found. *D. anomalus* has been rarely reported as a major yeast in milk kefir. The absolute contribution of this species to milk kefir microbial community was reported by Garofalo et al. (2015) when studied six Italian milk kefirs. Interestingly, *D. anomalus* was only detected in the Italian kefir sample (EK) and in none of the German samples. It seems that all samples contain lactose fermenting and non-lactose fermenting yeasts, a factor that probably stabilizes yeast communities in kefir. This character of yeast communities was found in all kefir samples in this study, and was also reported by Garofalo et al. (2015).

In some of the grain fractions, the detection of some species, i.e., *Ln. mesenteroides*, *Lc. lactis*, *A. fabarum* and *A. orientalis* among bacteria and *Kz. unispora* among yeast species, either was not achieved at all or they were lower than LOQ (Table 4), and accordingly rated as being negative. While these species have been identified in a high number in beverage fractions, their low number in grain fractions is probably due to their release from the grain structure during the washing steps. This might also indicate that they do not bind strongly to the grain structure or other microbial consortia’s members. As the absence of these species in kefir grain vs. kefir beverage was only observed in some kefirs and not in all cases (e.g., detection of *A. orientalis* in PN vs. FN kefirs as an example), this may imply that binding properties is strain-specific. Cell surface-related phenomena like cell-cell and cell-matrix adhesion, flocculation, auto- and co-aggregation are not only strain-specific (Soares, 2011; Wang et al., 2012), but also strongly correlated to medium composition, e.g., bivalent ions strength and the pH-value (Wang et al., 2019). A high concentration of calcium and a low pH-value have been related with higher flocculation of different bacteria and yeasts (Soares, 2011; Wang et al., 2017, 2019). Accordingly, it can be implied that a natural pH-value of our washing solution and the presence of monovalent ions (Na^+^) instead of divalent ions (which are naturally provided by milk) can lead to resolve some cell-cell attachments. Although the effect of medium composition and pH-value on cell attachments have been studied for a few numbers of *Lactobacills* species (Wang et al., 2019), no kefir-related microorganisms were a study target though. It is also worthwhile to mention that the arrangement of microbial species on or in a grain structure is still a matter of research. Although some studies show that the microorganisms occupy all interior and exterior surface of grains (Magalhães et al., 2010a, Magalhães et al., 2011; Wang et al., 2012), microorganisms are hardly observed on the outer surface of the grains, but only embedded in the fibrillar matrix near the surface in another study (Ismaiel et al., 2011). There is also the possibility that this property depends on the microbial community composition and varies among different kefirs harboring different species and strains, which open a topic for further studies.

## Conclusion

This study presents new multiplex TaqMan qPCR assays that can detect and quantify eleven frequently reported bacteria and yeast species of milk kefir microbiota. Due to its relatively fast nature, the method can be an advantageous tool when the dynamics of these species in microbial communities shall be monitored. Furthermore, by adjustment of the reporter dyes, it is possible to adapt different custom-based multiplex assays. Based on the results of this study, *Lb. kefiranofaciens* and *Lb. kefiri* are ubiquitous in milk kefirs, though the presence of other nine species varied among different kefirs.

## Data Availability Statement

The datasets presented in this study can be found in online repositories. The names of the repository/repositories and accession number(s) can be found in the article/ Supplementary Material.

## Author Contributions

FN contributed to design of the experiment and analysis, and writing the original draft. PN, JK, and SJ contributed to the writing, reviewing, and editing the manuscript.

## Conflict of Interest

The authors declare that the research was conducted in the absence of any commercial or financial relationships that could be construed as a potential conflict of interest.

## Footnotes

1 https://blast.ncbi.nlm.nih.gov/Blast.cgi

2 http://scienceprimer.com/copy-number-calculator-for-realtime-pcr

3 www.ncbi.nlm.nih.gov
