# Supplementary material for: Quantification of Major Bacteria and Yeast Species in Kefir Consortia by Multiplex TaqMan qPCR
Source: Front Microbiol. 2020 Jun 16;11:1291. doi: 10.3389/fmicb.2020.01291 (PMC7315786; doi:10.3389/fmicb.2020.01291)
Supplement: Supplementary file 1 [file Data_Sheet_1.docx]

**A.**

**Supplementary Figure 1.** Multiplex conventional PCR using primers sets designed in this study for A) bacterial targets; lanes 1-7 and nontargets; lanes 8-13 and B) Yeasts targets; lanes 14-18 and nontarget; lane 19, M 100bp Ladder. 1, Lb. kefiranofaciens, 2, Lb. kefiri, 3, A. orintalis, 4, A. fabarum, 5, Ln. mesenteroides, 6, Lc. lactis ssp. lactis, 7, Lc. lactis ssp. cremoris, 8, Lb. helveticus, 9, Lb. reuteri, 10, Lb. paracasei, 11, Lb. parakefiri, 12, Lb. plantarum, 13, W. cibaria, 14, S. cerevisiae, 15, D. anomalous, 16, Kz. unispora, 17, Kl. marxianus, 18, Kz. turicensis and 19, Kz. exigua.


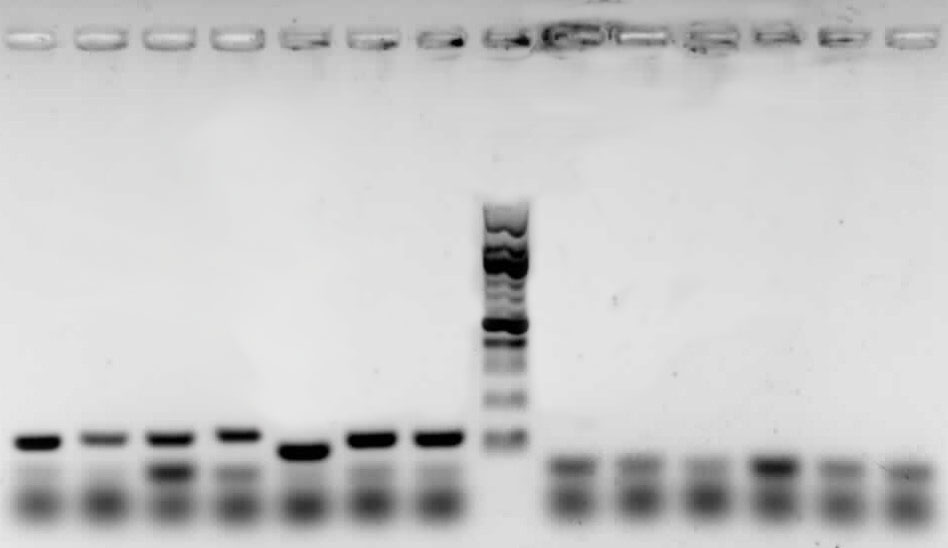


**1 2 3 4 5 6 7 M 8 9 10 11 12 13**

**500**

**300**

**200**

**100**


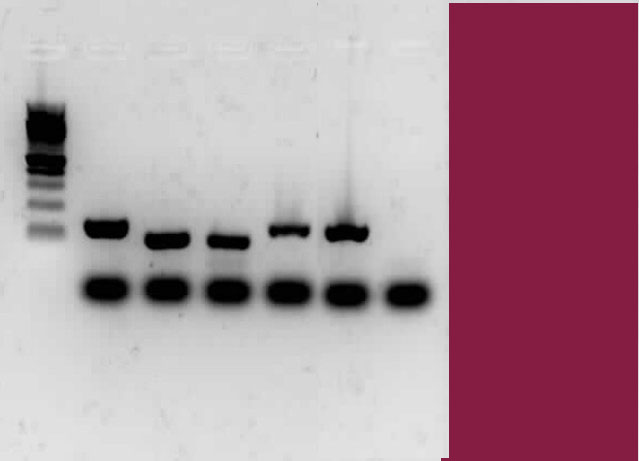


**M 14 15 16 17 18 19**

**100**

**500**

**200**

Figure S1.

Multiplex conventional PCR using primers sets designed in this study for A) bacteria; targets, lane 1-7 and nontargets lane 8-13 and B) Yeasts; targets, lane 14-18 and nontarget, lane 19, M 100bp

**B.**

**Figure S1**:

| A  C  B 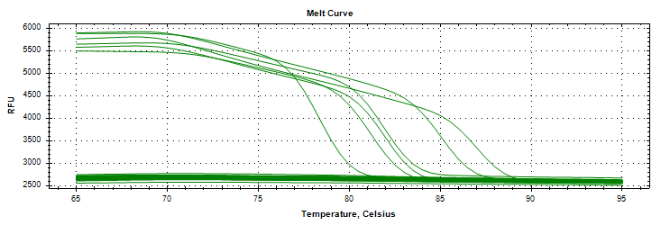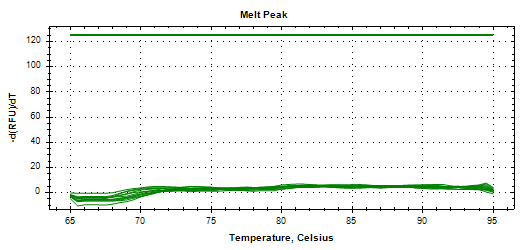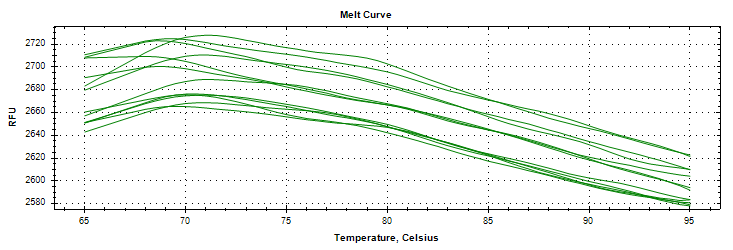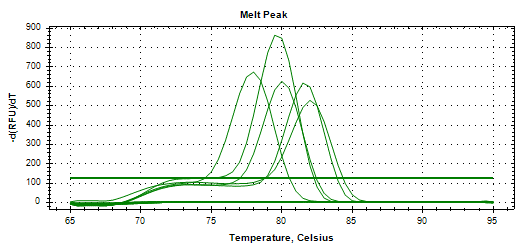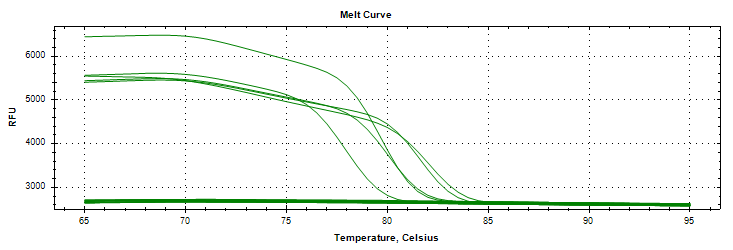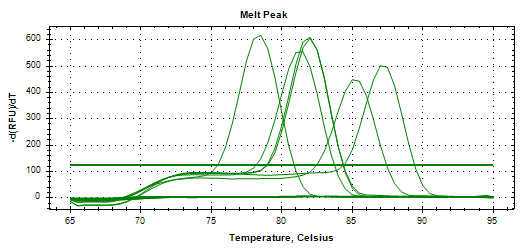 |  |
| --- | --- |

**Supplementary Figure 2.** Melt curve analysis in simplex qPCR runs; (A) without target DNA, (B) with bacterial target DNA (peaks from left to right correspond to Ln. mesenteroides, Lb. kefiranofaciens, Lb. kefiri, Lc. lactis, A. orientalis and A. fabarum) and (C) with yeast target DNA ((peaks from left to right correspond to Kz. unispora, Kz. turicensis, Kl. marxianus, S. cerevisiae and D. anomalous).

| 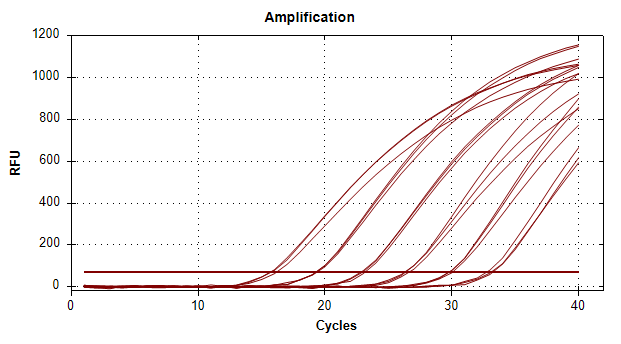 | 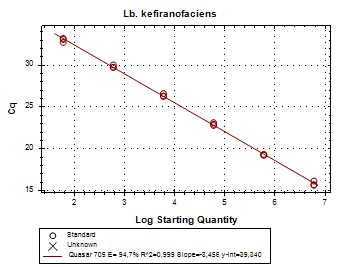 |
| --- | --- |
| 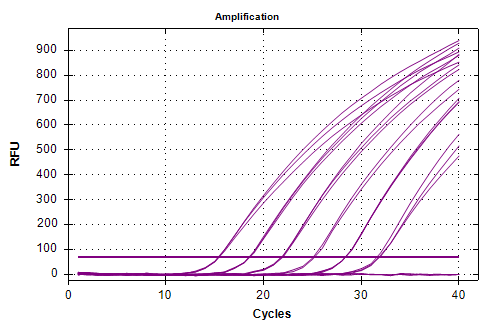 | 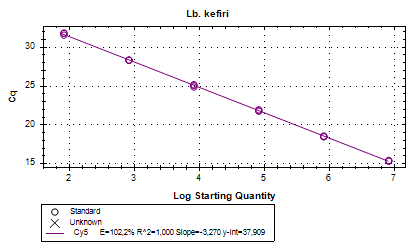 |
| 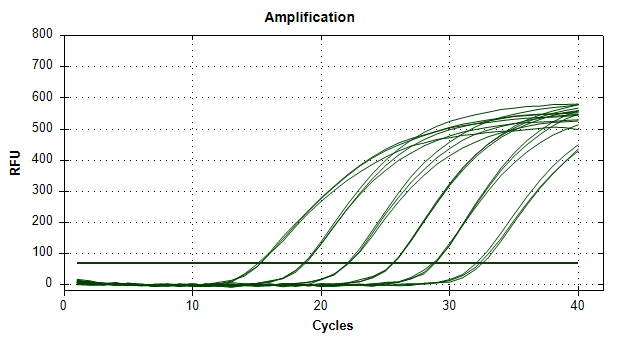 | 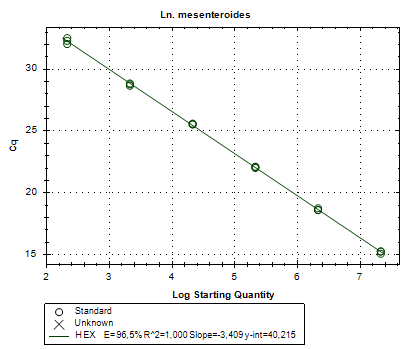 |
| 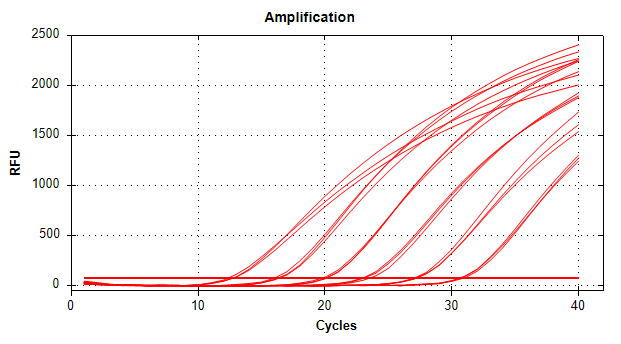 | 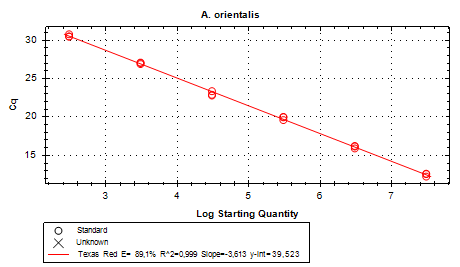 |
| 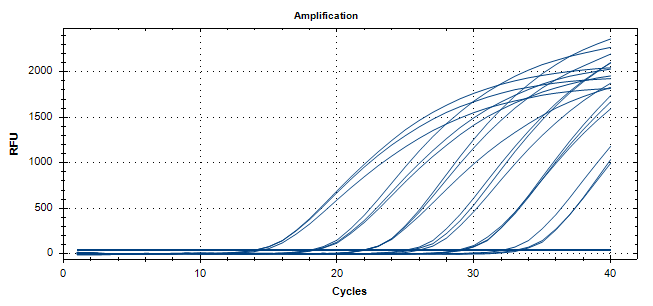 | 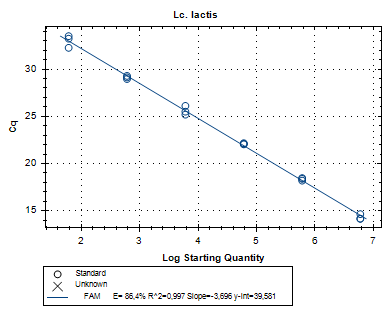 |
| 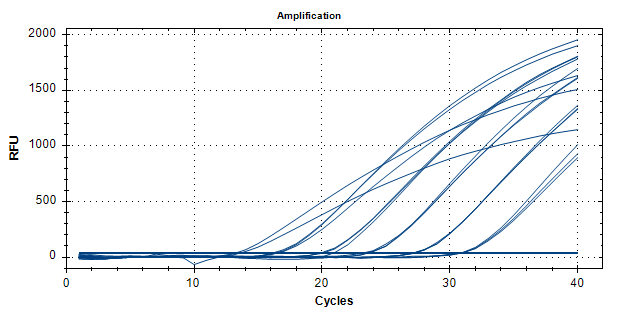 | 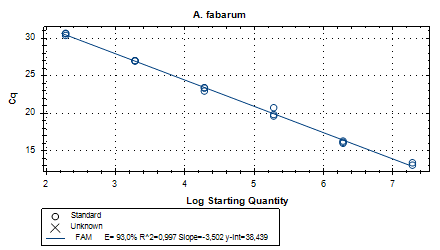 |
|  | |

**Supplementary Figure 3.** Analysis of ten-fold serially diluted gDNA extracted of six target bacteria species in simplex qPCR runs; first column: amplification plot and second column; the linear relationship between quantification cycle (Cq) and log DNA copy number per reaction.

| 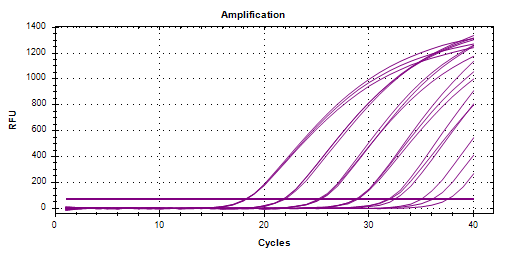 | 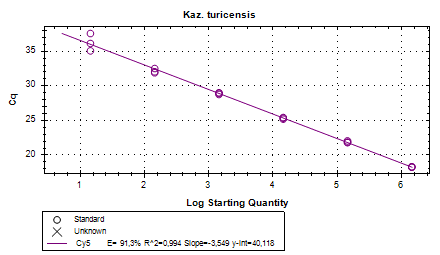 |
| --- | --- |
| 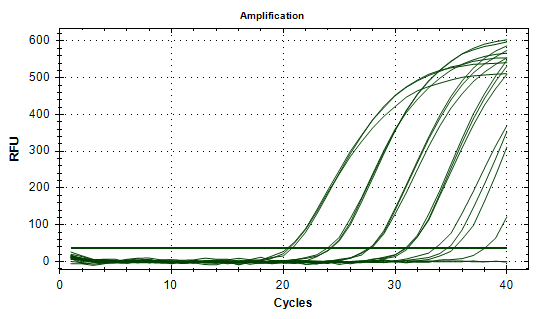 | 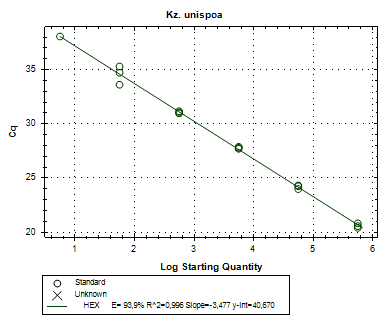 |
| 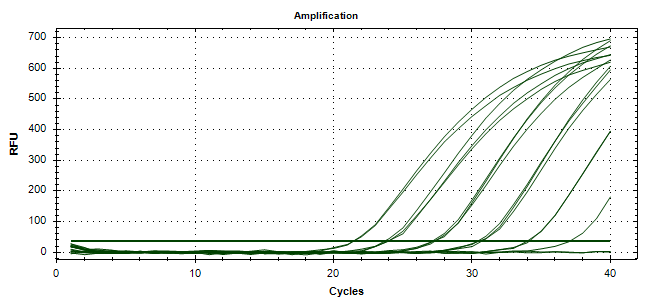 | 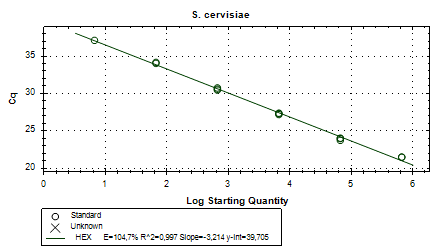 |
| 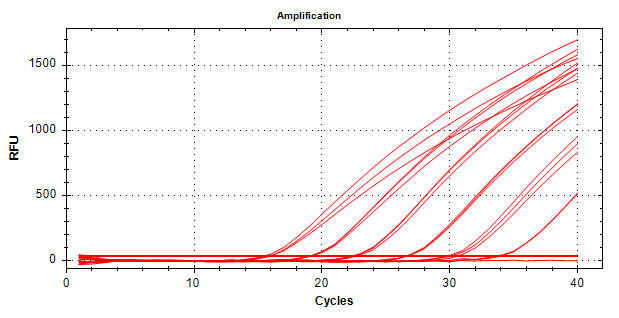 | 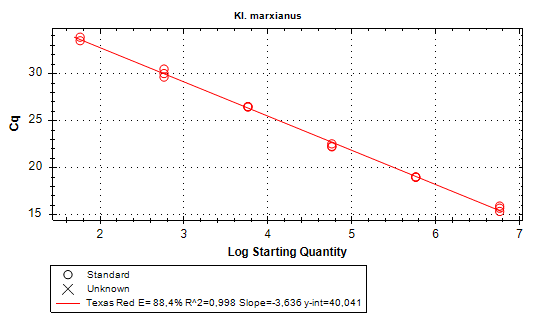 |
| 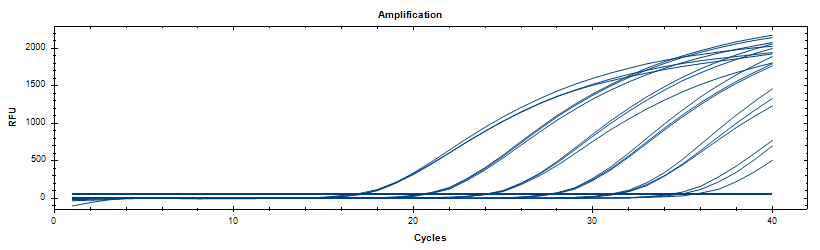 | 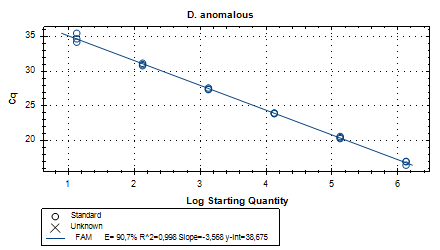 |
|  | |

**Supplementary Figure 4.** Analysis of ten-fold serially diluted gDNA extracted of five target yeast species in simplex qPCR runs; first column: amplification plot and second column; the linear relationship between quantification cycle (Cq) and log DNA copy number per reaction.

| 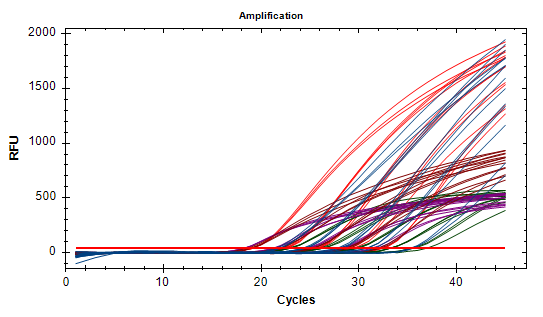 |  | 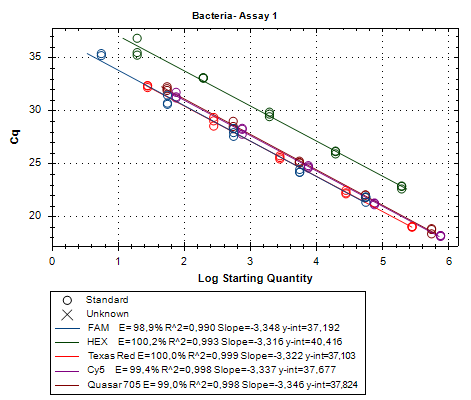 |
| --- | --- | --- |
| 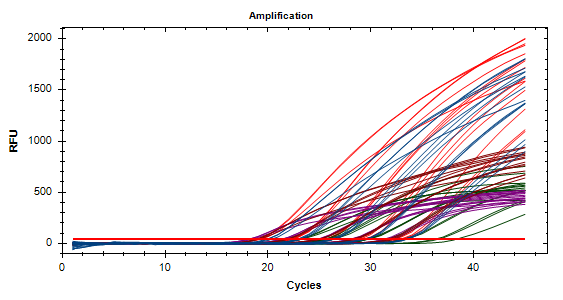 |  | 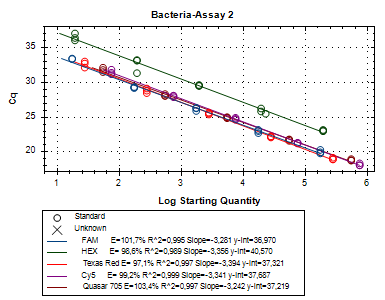 |

**Supplementary Figure 5.** Quantification of target bacteria species in multiplexed qPCR assays 1 and 2. First column; amplification curves using 10-fold serial dilutions of complete microbial genomic (CMG) as template and second column; the linear relationship between quantification cycle (Cq) and log DNA copy number per reaction. Assay 1 contain the probes for bacterial targets Lb. kefiranofaciens (Quasar 705), Lb. kefiri (Cy5), Ln. mesenteroides (HEX), A. oriantalis (Texas Red) and Lc. lactis (FAM) and assay 2 contain the probes for bacterial targets (Quasar 705), Lb. kefiri (Cy5), Ln. mesenteroides (HEX), A. oriantalis (Texas Red) and A. fabarum (FAM).

| 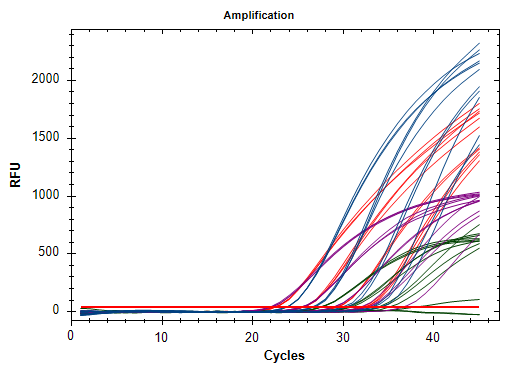 | 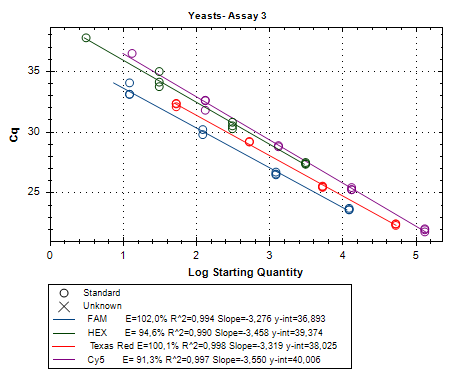 |
| --- | --- |
|  |  |
| 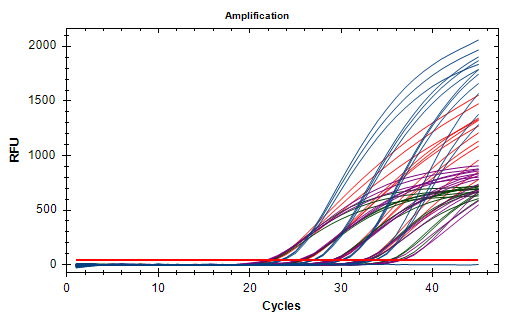 | 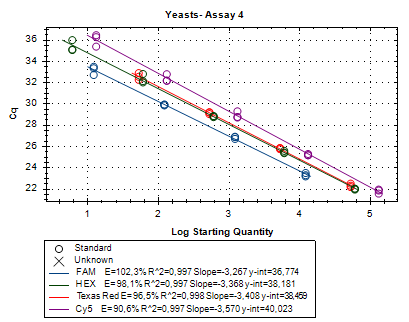 |

**Supplementary Figure S6.** Quantification of target bacteria species in multiplexed qPCR assays 3 and 4. First column; amplification curves using 10-fold serial dilutions of complete microbial genomic (CMG) as template and second column; the linear relationship between quantification cycle (Cq) and log DNA copy number per reaction. Assay 3 contains the probes for yeast targets Kz. turicensis (Cy5), Kz. unispora (HEX), Kl. marxianus (Texas Red) and D. anomalous (FAM) and assay 2 contains the probes for yeast targets Kz. turicensis (Cy5), S. cerevisiae (HEX), Kl. marxianus (Texas Red) and D. anomalous (FAM).

Supplementary Table 1. Quantity and purity of kefir grains’ DNA extracted by using two commercial kits

|  | FN grain | | PN grain | |
| --- | --- | --- | --- | --- |
|  | DNeasy PowerSoil Pro | DNeasy PowerBiofilm | DNeasy PowerSoil Pro | DNeasy PowerBiofilm |
| DNA yield ^a^ | 16.5 | 5.1 | 21.1 | 7.9 |
| DNA purity  A260/280 | 2.28 | 2.62 | 1.89 | 1.92 |
| A260/230 | 1.05 | 0.26 | 1.49 | 0.77 |
| ^a^ µg mL^-1^ | | | | |
